# Renal malakoplakia presenting as a renal mass in a 55-year-old man: a case report

**DOI:** 10.1186/1752-1947-6-379

**Published:** 2012-11-06

**Authors:** Maryam Abolhasani, Azam Mohammad Jafari, Mojgan Asgari, Hormoz Salimi

**Affiliations:** 1 Oncopathology Research Center, Tehran University of Medical Sciences (TUMS), Tehran, Iran; 2 Hasheminejad Clinical Research Developing Center (HCRDC), Tehran University of Medical Sciences (TUMS), Tehran, Iran; 3 Pathology Department, Hasheminejad Kidney Center, Valinejad Alley, Valiasr Avenue, Tehran, Iran; 4 Urology Department, Hasheminejad Kidney Center, Valinejad Alley, Valiasr Avenue, Tehran, Iran

## Abstract

**Introduction:**

Malakoplakia is an uncommon chronic inflammatory condition that has a gross and microscopic appearance resembling that of xanthogranulomatous pyelonephritis. It is characterized by distinctive Michaelis-Gutmann bodies. Malakoplakia can affect any organ system but genitourinary tract involvement is the most common, particularly in immunocompromised individuals. Very rare cases have been reported to present as a unifocal lesion mimicking a renal tumor.

**Case presentation:**

We report a case of renal malakoplakia in a 55-year-old Iranian man with a past history of recurrent urinary tract infections who presented with left flank pain. An ultrasound study showed a large solid left renal mass, and he underwent a left radical nephrectomy with a clinical diagnosis of a renal tumor. Pathology slides revealed the diffuse infiltration of sheets of Periodic Acid Schiff-positive histiocytes in his renal parenchyma; these cells showed strong immunoreactivity for CD 68. The final diagnosis was renal malakoplakia.

**Conclusion:**

Renal malakoplakia must be kept in mind for patients presenting with a renal mass and a history of long-term recurrent renal infections or renal failure. The large, rapidly growing nodules of malakoplakia may mimic renal cell carcinoma in imaging studies. In these cases, a true cut needle biopsy may help the correct diagnosis and prevent unnecessary surgery.

## Introduction

Michaelis and Gutmann first described malakoplakia in 1902 [1]. The term malakoplakia is derived from the Greek words *malakos*, which means soft, and *plakos*, which means plague [2]. The age at diagnosis ranges from 6 to 85 years, with an average age of 50 years at presentation. There is a female predominance, with a female to male ratio of 4:1 [3]. Malakoplakia can affect any organ system including the gastrointestinal system, bones, lungs, lymph nodes and skin, but the collecting system of the urinary tract is most frequently involved. The renal lesion is most often multifocal [1].

Malakoplakia is associated with urinary tract infections in the majority of cases. Renal failure is common at presentation with variable severity [4]. About 40% of patients have some form of immunosuppression, including solid organ transplants, autoimmune diseases requiring steroid use or chemotherapy, chronic systemic diseases, malignancy, alcohol abuse and poorly controlled diabetes mellitus [5]. Gram-negative bacteria are often associated with malakoplakia. *Escherichia coli* and *Proteus mirabilis* are the most commonly identified etiologic agents. *E. coli* contributes to about two thirds of cases [6,7].

In imaging studies, the appearance of the affected kidney ranges from that of a normal kidney to an enlarged, nonfunctioning kidney. Commonly, multiple poorly defined renal lesions enlarging the kidney, and often involving both kidneys, are present. The renal lesions can distort the pelvis and calices but seldom cause obstruction. Perirenal extension and renal vein thrombosis have been reported. Focal renal lesions are usually poorly defined and hypoechoic on ultrasound study. Parenchymal calcification is rare. A unifocal renal lesion is uncommon and can resemble a necrotic renal cell carcinoma [1]. Differential diagnoses in radiologic studies include local abscess, granuloma, xanthogranulomatous pyelonephritis, lymphoma and multifocal primary or metastatic tumors [8].

Gross inspection of the lesion reveals soft tan-yellow homogenous plaques and confluent nodules that are usually less than 1cm in diameter but can range up to 3 to 4cm and replace large areas of renal parenchyma. Fibrosis is also prominent. On microscopy, renal malakoplakia is characterized by large collections of plump macrophages with relatively few lymphocytes and plasma cells. The distinctive basophilic inclusions with surrounding clear halos known as Michaelis-Gutmann bodies are found within both the histiocytes and extracellularly in the stroma. Michaelis-Gutmann inclusions on hematoxylin and eosin-stained slides are basophilic because of the stain’s affinity for the iron and perhaps calcium, which is eventually absorbed into the acid bacterial lipid polysaccharide substances. They are also Periodic Acid Schiff-positive [9]. The microscopic differential diagnoses are Whipple’s lipodystrophy, xanthogranulomatous pyelonephritis, megalocytic interstitial nephritis and granular cell tumor [1].

The diagnosis of malakoplakia must be kept in mind for patients presenting with a renal mass and a history of long-term recurrent renal infections or renal failure [4]. Renal malakoplakia may mimic renal tumors and lead to unnecessary surgery. The patient in our report had renal malakoplakia but underwent a nephrectomy with the clinical diagnosis of a renal tumor. A nephrectomy can be a choice for unifocal malakoplakia, but the preoperative diagnosis of renal malakoplakia in appropriate clinical settings can prevent unnecessary surgery.

## Case presentation

**Figure 1:**
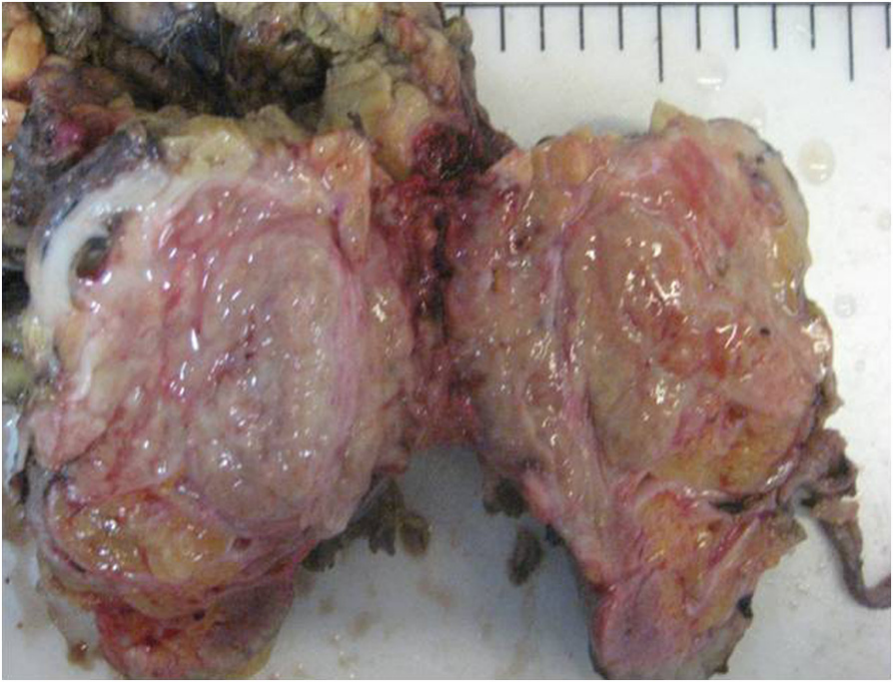
**Gross examination of the kidney.** A yellowish rubbery solid mass occupied the superior and middle poles with extension to the renal sinus and perinephric fat.

A 55-year-old Iranian man was hospitalized due to left flank pain for five years before admission as well as dysuria, urinary frequency, and a decrease of urinary force and caliber. He had a negative history of diabetes mellitus, hypertension, cardiovascular disease, malignancy and drug abuse. He mentioned a history of recurrent febrile urinary tract infections, which had been treated medically. On admission he was ill-looking. His temperature was 38.6°C. His blood pressure was 120/80mmHg. His respiratory rate was 16 breaths per minute and his pulse rate 83 beats per minute. Lung, cardiovascular, genital, rectal and neurological examinations were normal. His blood test results at admission showed a white blood cell count of 8200/μL, hemoglobin 14.5g/dL and platelet count of 233,000/μL. A urine analysis and culture showed no infection. An ultrasound study of his left kidney revealed a hypoechoic heterogeneous mass in the upper pole measuring 62×54mm. The clinical impression was a renal tumor or xanthogranulomatous pyelonephritis. He underwent a left radical nephrectomy. During the operation, the mass was seen attached to his descending colon and psoas muscle. The specimen was sent to the pathology ward. Gross examination showed an ill-defined yellowish rubbery solid mass, measuring 7×7×3cm, that occupied the superior pole and mid portion of his kidney (Figure 1). Macroscopic examination had revealed that the lesion extended to the renal sinus and perinephric fat.

**Figure 2:**
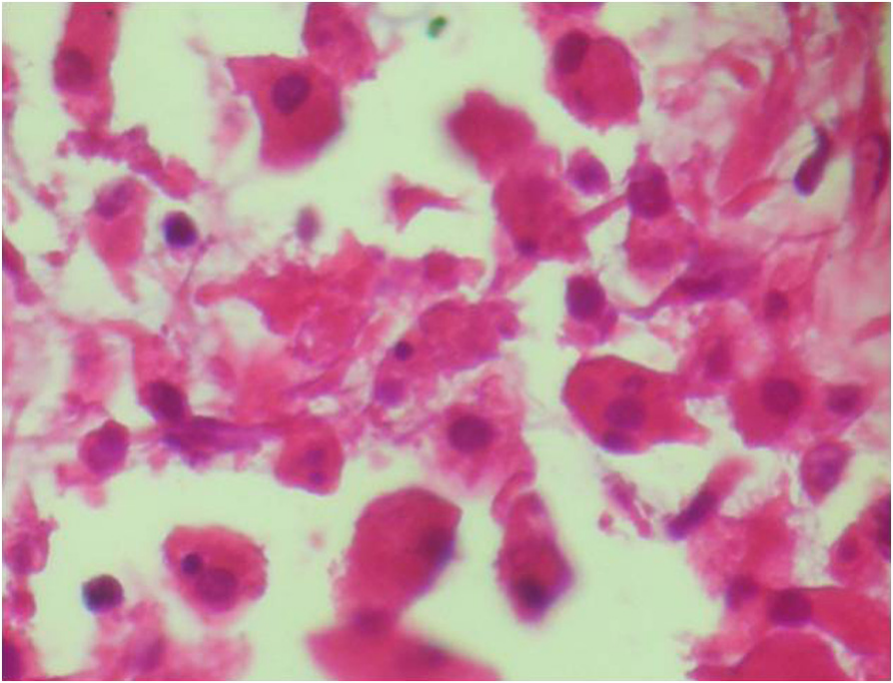
**Histological examination of the kidney (hematoxylin and eosin stain, ×40).** Infiltration of sheets of histiocytes in the renal parenchyma is noted.

**Figure 3:**
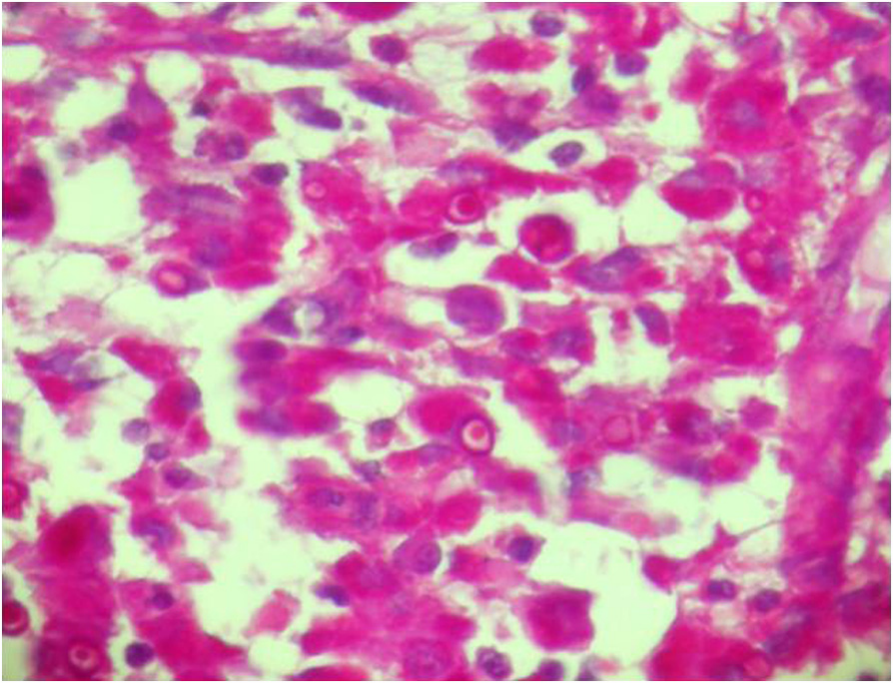
**Histological examination of the kidney (Periodic Acid Schiff stain, ×20).** Histiocytes with round concentric layered intracytoplasmic Michaelis-Gutmann bodies.

**Figure 4:**
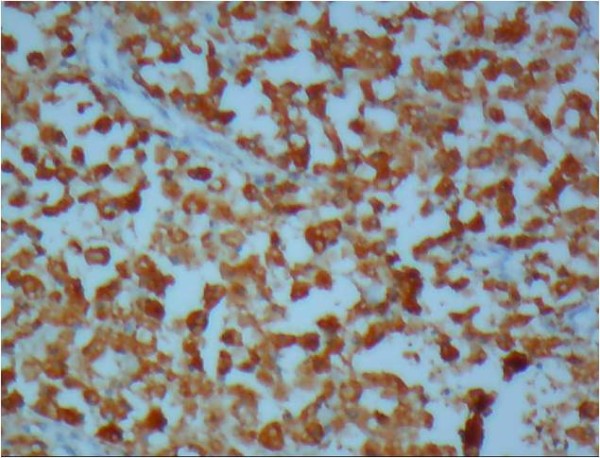
**Immunohistochemical staining for CD 68 (×10).** Strong reactivity for CD 68 is noted in the infiltration of histiocytes.

Histological examination revealed diffuse infiltration of sheets of Periodic Acid Schiff-positive histiocytes into the renal parenchyma. These cells had granular acidophilic cytoplasms (Figure 2) and some of them showed round concentric layered intracytoplasmic Michaelis-Gutmann bodies (Figure 3). The histiocytes had infiltrated the renal sinus and perinephric fat. The immunohistochemical stainings showed strong reactivity for CD 68 (Figure 4), and vimentin and negative immunoreaction for pancytokeratin, CD 10, cytokeratin 7, E-cadherin, CD 15, α-methylacyl-coenzyme A racemase and tyrosine-protein kinase Kit (C-kit). The final diagnosis was renal parenchymal malakoplakia.

## Discussion

The patient in our case had a history of recurrent febrile urinary tract infections and the presence of a large unifocal mass in ultrasound study, which is uncommon for renal malakoplakia [1] and led to a clinical impression of a renal tumor or xanthogranulomatous pyelonephritis. The resected mass was grossly similar to a renal mass, and the light yellowish tan color of the lesion was more like a chromophobe renal cell carcinoma. The mass revealed perirenal and renal sinus extension in gross examination, which has also been reported in other cases [1]. Hematoxylin and eosin-stained slides of the lesion were rather typical for the diagnosis of malakoplakia. Due to the rare presentation of renal malakoplakia as a renal mass, Periodic Acid Schiff-staining was performed to better observe the Michaelis-Gutmann bodies to rule out other benign conditions mentioned in the introduction [1]. Immunohistochemical stainings were also performed to rule out concomitant renal cell carcinoma and malakoplakia, as there was a case reported in the literature [10].

The treatment of malakoplakia depends on the extent of the disease and the underlying conditions of the patient. Patients with bilateral or multifocal diseases are most often treated with antibiotics such as quinolones. A cholinergic agonist, bethanechol chloride, is also used to supplement antibiotic regimens to correct the lysosomal defect. Surgical excision is the choice treatment of unifocal diseases [3].

In the English literature there was another reported case from Iran of renal malakoplakia presenting as a renal mass. The patient in that case was a 10-year-old boy who presented with a fever of an unknown origin and a huge right renal mass, mostly suspected as Wilm’s tumor. The diagnosis of malakoplakia was made by an open biopsy, and he was treated with bethanechol chloride, trimethoprim-sulfamethoxazole and ascorbic acid. He became afebrile using this regimen and had a normal-sized kidney with no detectable tumor in a follow-up ultrasound study [11]. Because this patient was a child and Wilm’s tumor was suspected, an open biopsy was indicated and an unnecessary surgery was prevented. In a case reported by Wielenberg *et al*. [1], a 37-year-old man had a cystic left renal mass revealed by imaging studies that resolved after treatment of a bacterial infection but subsequently enlarged as a complicated cystic lesion, so he underwent radical nephrectomy. Our patient had a solid renal mass and his age was 55, which is more common for renal cell carcinoma.

In a case reported by Kapasi *et al*. [12], a 67-year-old man presented with acute left loin pain. Imaging studies showed a cystic mass with a thickened, irregular wall within his left renal sinus. Fine needle aspiration helped as a preliminary diagnostic method and showed large foamy macrophages with Michaelis-Gutmann bodies, so the renal malakoplakia was diagnosed before surgery. This patient also showed malakoplakia as a cystic mass in contrast with our patient, who had a solid mass [12]. There are two reported cases of renal malakoplakia leading to renal failure. Diwakar *et al*. reported a case of renal malakoplakia causing acute renal failure [4]. Hegde and Coulthard reported another case of bilateral renal malakoplakia that caused end-stage renal failure in a five-year-old girl [5].

## Conclusion

In patients with a fever of an unknown origin, flank pain, a renal mass and a history of recurrent urinary tract infections, the differential diagnosis should include xanthogranulomatous pyelonephritis, renal malakoplakia, renal tuberculosis and renal cell carcinoma. The large rapidly growing nodules of malakoplakia may mimic renal tumors, especially when ulcerated or accompanied by lymph node involvement. This paper may alert surgeons in cases with a high suspicion of renal inflammatory disease accompanied by a renal mass on imaging studies that fine needle aspiration, a true cut needle or an open biopsy may help to correctly diagnose a treatable disease entity and prevent unnecessary surgery. Pathologists should also be alert to the possibility of concomitant renal cell carcinoma if present [3,10].

## Consent

Written informed consent was obtained from the patient for the publication of this case report and accompanying images. A copy of the written consent is available for review by the Editor-in-Chief of this journal.

## Abbreviation

CD: Cluster of differentiation.

## Competing interests

The authors declare that they have no competing interests.

## Authors’ contributions

HS performed the nephrectomy. MAb and MAs performed the histological examination of the kidney, and AMJ and MAb were major contributors in writing the manuscript. All authors read and approved the final manuscript.
